# Right paraduodenal hernia presenting with strangulated obstruction with intestinal malrotation: a case report

**DOI:** 10.1093/jscr/rjae311

**Published:** 2024-05-18

**Authors:** Hideharu Tanaka, Saki Mitsutomoe, Narutoshi Nagao, Shuji Komori, Tomonari Suetsugu, Yoshinori Iwata, Taku Watanabe, Chihiro Tanaka, Masahiko Kawai

**Affiliations:** Department of Surgery, Gifu Prefectural General Medical Center, 4-6-1, Noishiki, Gifu, Gifu 500-8717, Japan; Department of Surgery, Gifu Prefectural General Medical Center, 4-6-1, Noishiki, Gifu, Gifu 500-8717, Japan; Department of Surgery, Gifu Prefectural General Medical Center, 4-6-1, Noishiki, Gifu, Gifu 500-8717, Japan; Department of Surgery, Gifu Prefectural General Medical Center, 4-6-1, Noishiki, Gifu, Gifu 500-8717, Japan; Department of Surgery, Gifu Prefectural General Medical Center, 4-6-1, Noishiki, Gifu, Gifu 500-8717, Japan; Department of Surgery, Gifu Prefectural General Medical Center, 4-6-1, Noishiki, Gifu, Gifu 500-8717, Japan; Department of Surgery, Gifu Prefectural General Medical Center, 4-6-1, Noishiki, Gifu, Gifu 500-8717, Japan; Department of Surgery, Gifu Prefectural General Medical Center, 4-6-1, Noishiki, Gifu, Gifu 500-8717, Japan; Department of Surgery, Gifu Prefectural General Medical Center, 4-6-1, Noishiki, Gifu, Gifu 500-8717, Japan

**Keywords:** paraduodenal hernia, intestinal malrotation, strangulation, intestinal obstruction, surgery

## Abstract

A paraduodenal hernia is a rare cause of an internal hernia that may require massive bowel resection; prompt diagnosis and surgical treatment are essential. In cases of malrotation, strangulation may occur both inside and outside the hernial sac. Strangulation outside the hernial sac makes the preoperative diagnosis more difficult. Herein, we report a patient with a right paraduodenal hernia, intestinal malrotation, and strangulation outside the hernia. An 86-year-old woman was admitted to our hospital with abdominal pain. Enhanced computed tomography showed a closed-loop obstruction of the hypo-enhancing small bowel and absence of a horizontal duodenal leg. The patient underwent an emergency laparotomy and was diagnosed with strangulated bowel obstruction due to a right paraduodenal hernia and malrotation. The patient underwent resection of the ischemic ileum, closure of the hernial orifice, and repositioning of the intestine. The postoperative course was uneventful. The patient reported no abdominal discomfort after 7 months of follow-up.

## Introduction

The incidence of internal hernias is 0.2%–0.9% of all intestinal obstructions, of which congenital paraduodenal hernias (PDHs) account for 53% [1] [2]. PDHs can be divided into left and right PDHs. Right PDHs are less common than left PDHs with a 1:3 ratio [3]. PDHs occur due to incomplete rotation and fixation abnormalities of the primitive midgut during fetal development [1, 3]. PDH with strangulation is associated with high mortality rates (20%–50%) [4, 5]. Although prompt diagnosis and surgical treatment are essential, PDHs occasionally are a diagnostic difficulty owing to the rarity [5]. In cases of right PDHs with malrotation, strangulation of the intestinal tract may occur both inside and outside the hernial sac. Strangulation outside the hernia sac makes the preoperative diagnosis more difficult.

Herein, we describe a patient with a right PDH, intestinal malrotation, and strangulation outside the hernia.

## Case report

**Figure 1:**
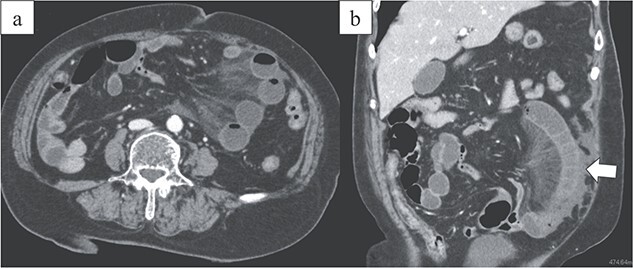
Images from enhanced CT. (a) Arterial-phase axial CT shows a hypo-enhancing small bowel in the left lower abdominal quadrant. (b) Arterial-phase coronal CT coronal enhanced CT shows a closed-loop obstruction of the hypo-enhancing small bowel (arrow).

**Figure 2:**
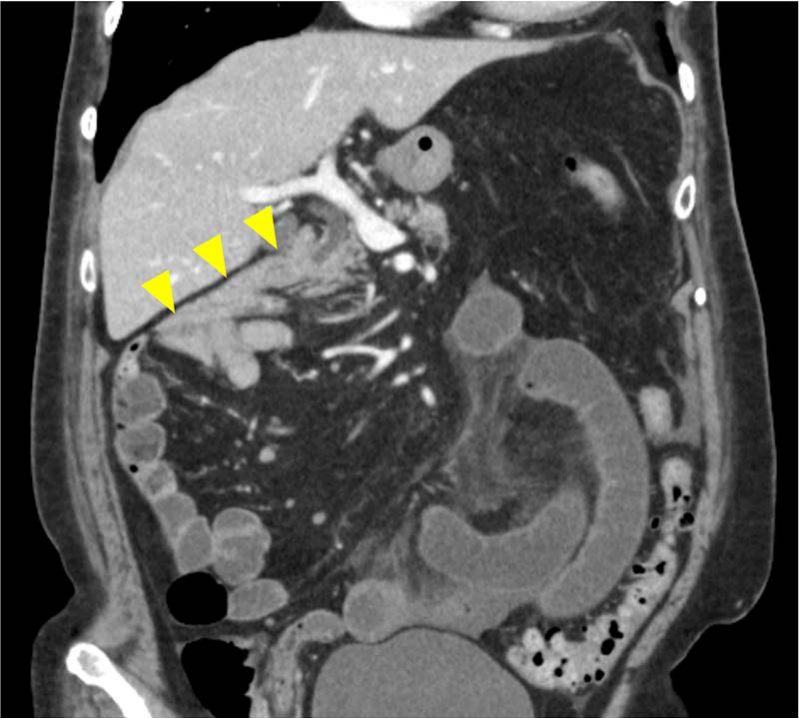
Image from enhanced CT. Contrast-enhanced CT shows the absence of a horizontal duodenal leg and the duodenum running caudally to the jejunum (arrowhead).

An 86-year-old woman with no prior abdominal surgeries was admitted to our hospital complaining of constant abdominal pain with an acute onset of 8 hr duration. The physical examination revealed tenderness of the lower abdomen. The laboratory test results were unremarkable, except for elevated lactate (3.0 mmol/l) and leukocyte counts (14 400/μl). Contrast-enhanced computed tomography (CT) revealed a closed-loop obstruction of the hypo-enhancing small bowel in the left lower abdominal quadrant, which suggested a strangulated bowel obstruction (Fig. 1). The duodenum did not form a horizontal limb and ran caudally to the jejunum (Fig. 2). The duodenojejunal flexure was abnormally located in the right upper abdominal quadrant, which suggested an intestinal malrotation. Sac-like capsulated small-bowel loops were barely visible.

A strangulated bowel obstruction with malrotation was suspected, and emergency surgery was performed.

**Figure 3:**
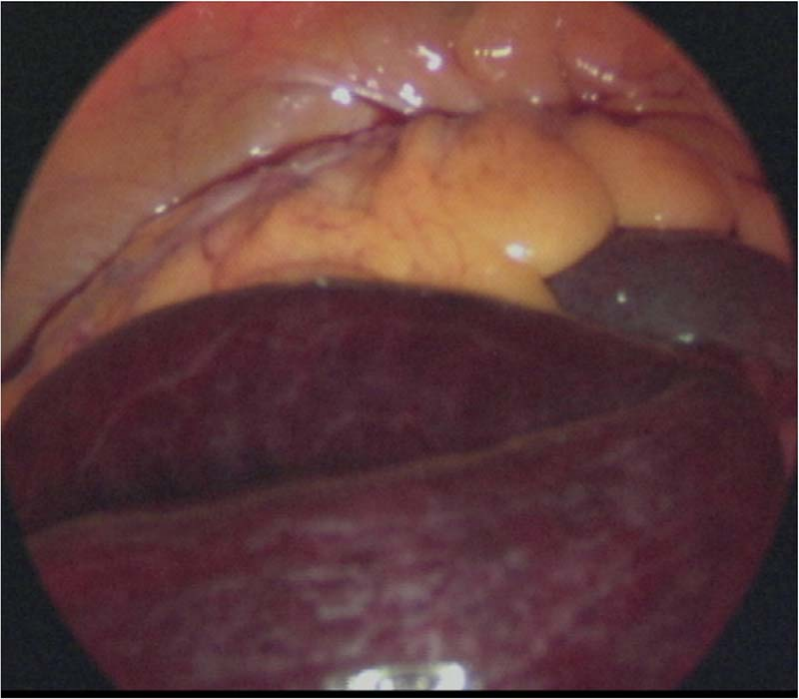
Intraoperative image. Laparoscopic examination of the abdominal cavity shows necrosis of the small bowel.

**Figure 4:**
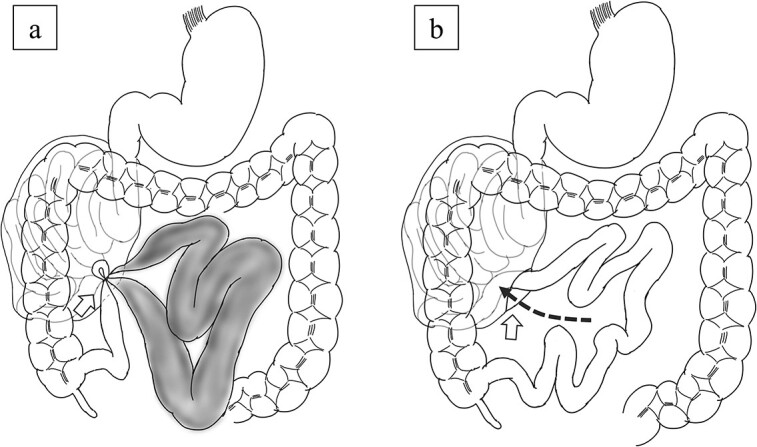
Scheme of the operative findings. (a) A 6-cm hernial orifice (arrow) was observed at the fusion defect between the right mesocolon and retroperitoneum. Intestinal necrosis was observed in 80 cm of the ileum within the abdominal cavity outside the hernial sac and no intestinal necrosis was observed in the small intestine within the hernial sac. (b) The jejunum had migrated to the inside of the hernial sac through the orifice (arrow).

**Figure 5:**
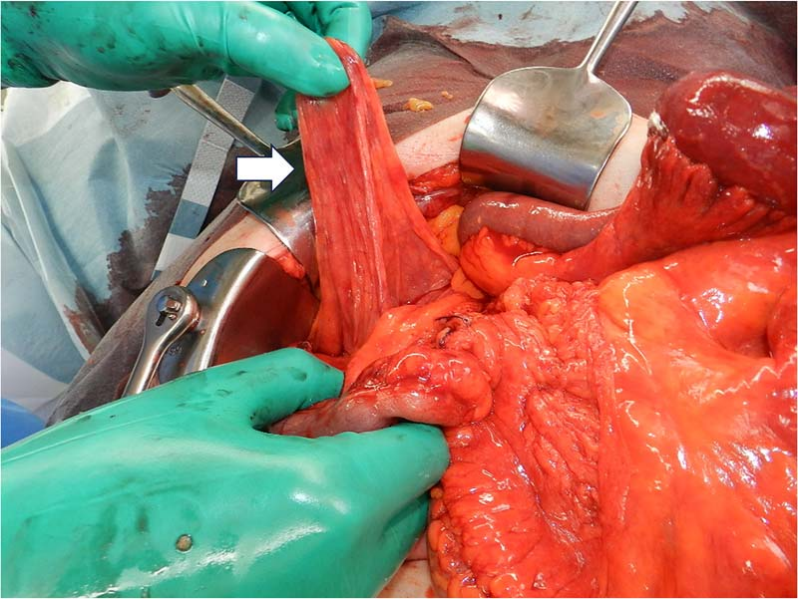
Intraoperative image showing that the hernial sac (arrow) was 12 × 6 cm in size.

**Figure 6:**
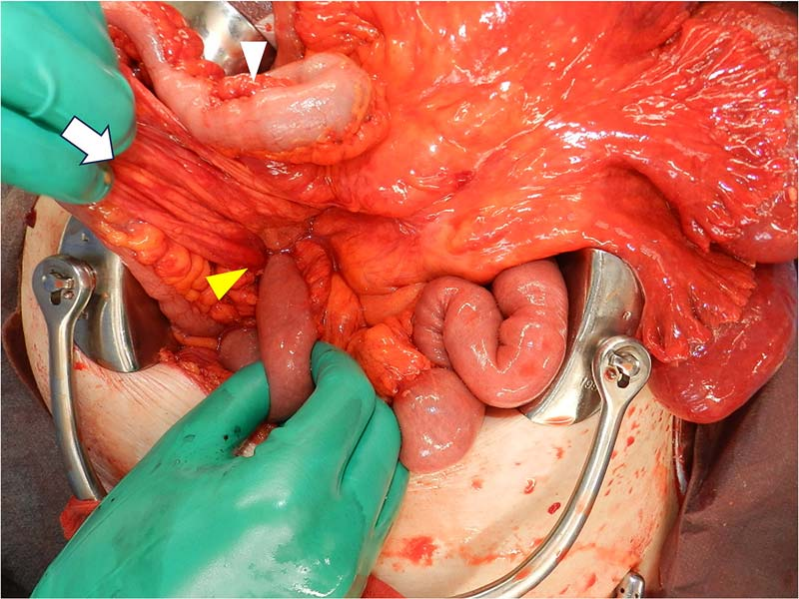
Intraoperative image after replacing the small bowel into the abdominal cavity shows that the duodenum did not form a horizontal leg and ran caudally from the descending leg in the hernial sac to the jejunum. Proximal jejunum, lower arrowhead; hernial sac, arrow; terminal ileum, upper arrowhead.

A laparoscopic approach was initiated, which revealed necrosis of the small bowel (Fig. 3). Because the intestinal dilatation was severe, the procedure was converted to a laparotomy to better visualize the abdominal cavity. The abdomen was opened through a median abdominal incision. Most of the small intestine was located on the dorsal aspect of the right mesocolon, and a 6-cm hernial orifice was noted at the fusion defect between the right mesocolon and retroperitoneum. Intestinal necrosis was observed in 80 cm of the ileum in the abdominal cavity outside the hernial sac. No intestinal necrosis was apparent in the small intestine within the hernial sac (Fig. 4a). The hernial sac was 12 × 6 cm in size (Fig. 5). The duodenum did not form a horizontal leg and ran caudally from the descending leg in the hernial sac to the jejunum (Fig. 6). There was no Ladd ligament or band formation. The patient was diagnosed intraoperatively with a strangulated bowel obstruction due to a right PDH with malrotation. Intestinal necrosis involved 80 cm of the ileum in the abdominal cavity outside the hernial sac. A partial resection of the necrotic jejunum was performed. After partial resection of the necrotic ileum, all intestines in the hernial sac were replaced in the abdominal cavity. The hernial sac was resected, and the hernial orifice was closed with suture. Because the ascending colon was in the normal anatomic position, no fixation procedure was performed. The total operative time was 197 min, and the estimated blood loss was 800 ml, including ascites fluid. The postoperative course was uneventful, and she was discharged on postoperative Day 10. The patient reported no abdominal discomfort after 7 months of follow-up.

## Discussion

A right PDH develops from embryonic anomalies of the peritoneum and is associated with abnormal intestinal malrotation [3, 6, 5]. Bill *et al.* [7] defined a right PDH as the IIC type, in which the cephalic midgut (duodenum and jejunum) stops rotating midway during the second stage of bowel rotation and the caudal midgut (ascending colon) rotates normally, resulting in most of the small bowel located dorsal to the mesentery of the ascending colon. Intestinal malrotation occurs in 1 in 10 000 live births with 80% of all cases developing symptoms within the first month of life [8]. Symptoms in adults are extremely rare, occurring in 0.2%–0.5% of all cases [9].

Upper-gastrointestinal contrast and CT findings facilitate the diagnosis. A typical finding of malrotation is the absence of a horizontal duodenal leg and leftward migration of the superior mesenteric vein relative to the superior mesenteric artery [10]. A right PDH is characterized by a dilated loop of small intestine that forms a localized cluster in the right upper abdomen and appears to be encased in a sac-like structure [10]. In our case, the ileum outside the hernia sac was strangulated and ischemic, whereas the jejunum inside the hernia sac was not ischemic. Therefore, abnormal findings in the hernial sac were not noticeable, and it was difficult to make an accurate preoperative diagnosis of a right PDH. On a CT scan obtained 4 years earlier, the jejunum was located in the abdominal cavity. It was thought that the jejunum had migrated to the inside of the hernial sac through the orifice and was strangulated (Fig. 4b). In cases of right PDH with malrotation, strangulation of the intestinal tract may occur both inside and outside the hernial sac, but strangulation outside the hernial sac is rare. Indeed, there are only three published case reports of a right PDH with malrotation and strangulation necrosis outside the hernia sac, including our case, except for volvulus [11, 12].

The principles of surgical management for a right PDH include repositioning of the fitted digestive tract, closure or widening of the hernial orifice, if any, and resection of the ischemic intestinal segment. The Ladd procedure, in which the small intestine is placed on the right abdominal side and the large intestine is on the left abdominal side, is also recommended, especially in children [13]. Wang *et al.* [14] summarized 34 patients with right PDHs that were confirmed surgically, in which Ladd procedure or closure procedure was performed in most cases. In recent years, reports on the usefulness of laparoscopic surgery have increased [6], and accurate preoperative diagnosis is important. No postoperative recurrences have been reported in previous cases with any of the surgical procedures. The surgical procedures may be selected according to the characteristics of each case [14, 15].

In conclusion, especially in cases of a right PDH with malrotation, strangulation of the intestinal tract may occur both inside and outside the hernial sac. When necrosis occurs outside the hernial sac, the findings inside the hernial sac may be inconspicuous, making the preoperative diagnosis difficult. When strangulated bowel obstruction is observed, the duodenal run and previous images should be confirmed, and prompt treatment should be given considering the possibility of intestinal malrotation.

## Data Availability

The data can be obtained by emailing the corresponding author.
